# Perceived efficacy of herbal remedies by users accessing primary healthcare in Trinidad

**DOI:** 10.1186/1472-6882-7-4

**Published:** 2007-02-07

**Authors:** Yuri N Clement, Jamie Morton-Gittens, Luke Basdeo, Alexander Blades, Marie-Joanna Francis, Natalie Gomes, Meer Janjua, Adelle Singh

**Affiliations:** 1 Department of Para-Clinical Sciences, Faculty of Medical Sciences, The University of the West Indies, St. Augustine, Trinidad and Tobago

## Abstract

**Background:**

The increasing global popularity of herbal remedies requires further investigation to determine the probable factors driving this burgeoning phenomenon. We propose that the users' perception of efficacy is an important factor and assessed the perceived efficacy of herbal remedies by users accessing primary health facilities throughout Trinidad. Additionally, we determined how these users rated herbal remedies compared to conventional allopathic medicines as being less, equally or more efficacious.

**Methods:**

A descriptive cross-sectional study was undertaken at 16 randomly selected primary healthcare facilities throughout Trinidad during June-August 2005. A *de novo*, pilot-tested questionnaire was interviewer-administered to confirmed herbal users (previous or current). Stepwise multiple regression analysis was done to determine the influence of predictor variables on perceived efficacy and comparative efficacy with conventional medicines.

**Results:**

265 herbal users entered the study and cited over 100 herbs for the promotion of health/wellness and the management of specific health concerns. Garlic was the most popular herb (in 48.3% of the sample) and was used for the common cold, cough, fever, as 'blood cleansers' and carminatives. It was also used in 20% of hypertension patients. 230 users (86.8%) indicated that herbs were efficacious and perceived that they had equal or greater efficacy than conventional allopathic medicines. Gender, ethnicity, income and years of formal education did not influence patients' perception of herb efficacy; however, age did (p = 0.036). Concomitant use of herbs and allopathic medicines was relatively high at 30%; and most users did not inform their attending physician.

**Conclusion:**

Most users perceived that herbs were efficacious, and in some instances, more efficacious than conventional medicines. We suggest that this perception may be a major contributing factor influencing the sustained and increasing popularity of herbs. Evidence-based research in the form of randomized controlled clinical trials should direct the proper use of herbs to validate (or otherwise) efficacy and determine safety. In the Caribbean, most indigenous herbs are not well investigated and this points to the urgent need for biomedical investigations to assess the safety profile and efficacy of our popular medicinal herbs.

## Background

The use of complementary and alternative medicines is burgeoning globally, especially in developed countries. However, over 80% of the population in developing countries depend on traditional healing modalities, including herbal remedies, for health maintenance and therapeutic management disease [1]. As in other developing regions, herbal remedy use is common in the Caribbean [2,3] and Merritt-Charles et al [4] in Trinidad recently reported an 86% lifetime prevalence of use among surgical outpatients on the island. Other studies in Trinidad revealed high use among diabetics [5] and asthmatics [6]. More surprising was the 40% prevalence of use among public health sector physicians in Trinidad, where most indicated favourable outcomes [7].

Although many studies identified the increasing prevalence of herbal use throughout the world, only a few reported on how patients perceived the efficacy of this healthcare modality in specific diseases [8,9]. In these studies, herbal remedies were not considered as an entity on its own, but as a subset of complementary and alternative medicines.

We suggest that one of the major factors contributing to the increasing popularity of herbs in developed countries and the sustained use in developing countries is the perception that herbal remedies are efficacious, and in some cases more so than physician-prescribed allopathic medicines. This favourable level of perceived efficacy would support continued use, and in a significant number of patients, concomitant use with conventional allopathic medicines. This scenario, of concomitant herb-drug use, raises the growing public health concern of potentially harmful interactions.

Our study was designed to assess how users of herbal remedies accessing public health facilities throughout Trinidad rated the efficacy of herbs, on its own, compared with physician-prescribed conventional medicines and as herb-drug combinations. We also determined whether these patients informed their attending physician of their herbal remedy use, and if not, their reasons for nondisclosure.

## Methods

The study was descriptive and cross-sectional in design using a *de novo *pilot-tested questionnaire and respondents were chosen from selected public health centres in Trinidad. The study was approved by the Ethics Committee, Faculty of Medical Sciences, The University of the West Indies, Trinidad and Tobago.

Patients were recruited proportionally from across the island by quota sampling, as population densities were markedly different throughout. Sixteen primary healthcare facilities were randomly selected and convenient sampling was used to obtain the quota of patients (over 16 years of age) who confirmed their use of herbal remedies (current or previous). All participating patients signed their informed consent and the survey instrument was interviewer-administered.

The survey instrument assessed demographic details including gender, age, ethnicity, household income, education, area of residence; a brief medical history (illnesses, conventional drugs used, and compliance with medication); herbal use (when and why herbal therapies were used, perceived effectiveness); conventional medicines/herbal comparison; physician's awareness of patient use of herbal remedies and the patient's attitude toward herbal use. Additionally, we recorded the herbs used, their indications for use and modes of use.

A previous study of herbal users showed that "excellent/very good/and good" rating of herbal remedies were reported 84.5% of the time by these users [10]. This prevalence was used to calculate a minimum sample size of 202 for our study, with a desired level of precision of ± 0.05 and type I error of 0.05 with a confidence interval of 95%. We assumed that non-response would not be a critical factor in our study, as questionnaires were interviewer-administered.

Stepwise multiple regression analysis was used to determine the influence of predictor variables (demographics: gender, age, number of years of formal education, income and ethnicity; disease states: diabetes, hypertension and asthma) on perceived efficacy and comparative efficacy with conventional medicines. The influence of abovementioned predictor variables on disclosure of herbal remedy use to physician was also analyzed. Predictor variables not contributing to the model were systematically removed in the stepwise regression process. The data was analyzed using the SPSS program for Windows (Version 13.0, Chicago, IL).

## Results

Two hundred and sixty-five (265) patients agreed to enter the study, Table 1. Interviewees were more likely to be over 46 years of age (56.7%), Asian Indian (45.3%), female (73.2%), with an annual household income of less than US$10,000, (84.1%), and with less than seven years of formal education (49.1%). Patients attended these primary health care facilities for various health conditions, but the most common reasons were for chronic disease management including hypertension (28%), diabetes mellitus (27%) and asthma (5%). They were treated with standard drugs and 41% of these patients indicated that they were compliant with the physician-prescribed medication.

**Table 1:** Demographic details of patient sample

**Demographic Data**	**Number (%)**
Age Group	
16 – 30	49 (18.5)
31 – 45	65 (24.5)
46 – 60	73 (27.6)
Over 60	76 (28.7)
Non Response	2 (0.76)
**Gender**	
Male	71 (26.8)
Female	194 (73.2)
**Annual Income ($US)**	
< 3,999	132 (49.8)
4,000 – 9,999	91 (34.3)
10,000 – 14,999	13 (4.9)
> 15,000	9 (3.4)
Non Response	20 (7.6)
**Years of formal education**	
None	19 (7.2)
≤ 7 years	121 (45.7)
> 7 years ≤ 12 years	94 (35.5)
> 12 years	25 (9.4)
Non Response	6 (2.3)
**Ethnicity**	
African	69 (26.0)
Asian Indian	120 (45.3)
Mixed	74 (27.9)
Other	1 (0.4)
Non Response	1 (0.4)

Over 100 herbs were cited in the study, and garlic was the most popular with almost half of the sample (48.3%) indicating its ethnobotanical usefulness as a carminative, in the management of hypertension and for general health and wellness (Table 2). About half of the sample (49.4%) reported that their herbal remedy use was for general health and wellness, while 112 (42.3%) self-administered herbs to treat specific illnesses and diseases, and 22 (8.3%) did not give any reason for herbal use. Many of the herbs were used to treat minor ailments including the common cold, cough and fever. Hypertension was the only chronic disease that featured with significant herbal remedy use, where 20% of patients used garlic (Table 3). Most patients (203 of 265; 76.6%) did not inform the attending physician regarding their herbal remedy use because they thought that it was insignificant (117 of 203; 57.6%) or could not recall being specifically asked about it (56 of 203; 27.6%).

**Table 2:** Common medicinal plants and their ethnobotanical use, ranked by prevalence.

**Common name**	**Botanical name**	**Ethnobotanical Use**	**n (%)**
Garlic	*Allium sativum *L.	Carminative, hypertension	128 (48.3)
Orange (rind)	*Citrus sinensis (*L.) Osbeck	Carminative, general health, hypertension, menstrual pain	109 (41.1)
Ginger	*Zingiber officinale *Roscoe	Carminative, common cold, cough, general health	100 (37.7)
Lemongrass	*Cymbopogon citratus *(D.C.) Stapfl	Fever, common cold, cough, general health	92 (34.7)
Aloes	*Aloe vera (Aloe barbadensis *Miller)	'blood cleanser', anticoagulant, general health	70 (26.4)
Shandileer	*Leonotis nepetifolia *(L.) R.Br.	Common cold, cough, fever	53 (20.0)
Zebapique	*Neurolaena lobata*	Common cold, cough, fever, diabetes mellitus	32 (12.1)
Christmas bush	*Chromolaena odorata *(L.) R.M. King & H. Rob	Common cold, cough	31 (11.7)
Vervine	*Stachytarpheta jamaicensis *(L.) Vahl	'blood cleanser', common cold, general health, lactation	24 (9.1)
Karaili	*Momordica charantia *L.	Diabetes mellitus, 'blood cleanser', gripe	22 (8.3)
Noni	*Morinda citrifolia *Linn.	General health, diabetes mellitus	17 (6.4)
Shining bush	*Peperomia pellucida *(L.) Kunth	'blood cleanser', common cold, general health	16 (6.0)
Lime (young buds)	*Citrus limetta *Risso	Common cold, general health	15 (5.7)
Bois cano	*Cecropia peltata *L.	Common cold, cough, general health	14 (5.3)
Black sage	*Cordida cylindristachya *R.S.	Common cold, cough	14 (5.3)
Shadon beni	*Eryngium foetidum *L.	Common cold, fever, pain	14 (5.3)
Wonder-of-the-world	*Kalanchoe pinnata *(Lam.) Pers.	Pain	14 (5.3)
Wild senna	*Senna alata *(L.) Roxb	'Colon cleanser'	13 (4.9)

**Table 3:** Common health concerns for self-medication with herbal remedies, ranked by prevalence

**Health use**	**Number of herbs cited**	**Most commonly cited herbs**	**n (%)**
Colds and coughs	42	Shandileer (*Leonotis nepetifolia*)	68 (25.7)
		Ginger (*Zingiber officinale*)	33 (12.5)
		Christmas bush (*Chromolaena odorata*)	30 (11.3)
		Lemongrass (*Cymbopogon citratus*)	29 (10.9)
		Zebapique (*Neurolaena lobata*)	29 (10.3)
'Blood cleanser'	29	Aloes (*Aloe vera; Aloe barbadensis*)	53 (20.0)
General health and wellness	25	Ginger (*Zingiber officinale*)	20 (7.6)
		Garlic (*Allium sativum*)	19 (7.2)
		Lemongrass (*Cymbopogon citratus*)	15 (5.7)
Hypertension	15	Garlic (*Allium sativum*)	53 (20.0)
Carminative	12	Orange rind (*Citrus sinensis (*L.) Osbeck)	88 (33.2)
		Garlic (*Allium sativum*)	60 (22.6)
		Ginger (*Zingiber officinale*)	46 (17.4)

Two hundred and thirty respondents (86.8%) perceived that their previous or current use of herbal remedies was efficacious, with only 26 (9.8%) reporting that herbal remedies were effective 'sometimes', Table 4. Only 9 (3.4%) believed that herbal remedy use was totally ineffective. Using stepwise multiple regression analysis a significant model emerged (F = 4.464, p = 0.036, adjusted R^2 ^= 0.014); where age was a significant predictor variable with B = 0.136 and p = 0.036. Gender, family income, ethnicity and number of years of formal education were not significant predictors of the patient's perception of efficacy of herbal remedies. The data was further analyzed to determine whether these demographic variables had any influence of patients' disclosure of herbal use to their physician; we found that none of these demographic variables were significant predictors in the stepwise regression model. Likewise, we subjected the data to regression analysis to determine the influence of disease states (hypertension, diabetes and asthma) on the perception of efficacy; disease state was not a significant predictor in the stepwise regression model.

**Table 4:** Patients' rating of efficacy of herbal remedies

**Demographic factor**	**n**	**Believes that herbal remedies are effective n (%)**		
		**Yes**	**No**	**Sometimes**
**Age Group**				
16 – 30	49	47 (95.9)	0 (0.0)	2 (4.1)
31 – 45	65	55 (84.6)	5 (7.7)	5 (7.7)
46 – 60	73	62 (84.9)	3 (4.1)	8 (11.0)
Over 60	76	64 (84.2)	1 (1.3)	11 (14.5)
Non Response	2			
		228 (86.0)	9 (3.4)	26 (9.8)
		**P = 0.036**		
**Gender**				
Male	71	63 (88.7)	0 (0.0)	8 (11.3)
Female	194	167 (86.1)	9 (4.6)	18 (9.3)
		230 (86.8)	9 (3.4)	26 (9.8)
		NS		
**Annual Income ($US)**				
< 3,999	132	114 (86.4)	7 (5.3)	11 (8.3)
4,000 – 9,999	91	76 (83.5)	2 (2.2)	13 (14.3)
10,000 – 14,999	13	13 (100.0)	0 (0.0)	0 (0.0)
> 15,000	9	7 (77.8)	0 (0.0)	2 (22.2)
Non Response	20			
		210 (79.3)	9 (3.4)	26 (9.8)
		NS		
**Years of formal education**				
None	19	19 (84.2)	1 (5.3)	2 (10.5)
≤ 7 years	121	99 (81.8)	7 (5.8)	15 (12.4)
> 7 years ≤ 12 years	94	85 (90.4)	1 (1.1)	8 (8.5)
> 12 years	25	24 (96.0)	0 (0.0)	1 (4.0)
Non Response	6			
		224 (84.5)	9 (3.4)	26 (9.8)
		NS		
Ethnicity*				
African	69	61 (88.4)	0 (0.0)	8 (11.6)
Asian Indian	120	97 (80.8)	9 (7.5)	14 (11.7)
Mixed	74	70 (94.6)	0 (0.0)	4 (5.4)
Other	1	1 (100.0)	0 (0.0)	0 (0.0)
Non Response	1			
		229 (86.4)	9 (3.4)	26 (9.8)
		NS		

* NS- not a significant predictor variable in stepwise regression model

Likewise, 86.6% believed that herbal remedies were equally or more efficacious than conventional medicines for specific ailments and diseases, and 26 (9.8%) believed that herbs were less efficacious, Table 5. Using stepwise multiple regression analysis no significant model emerged which indicated that gender, age, family income, number of years of formal education and ethnicity could not predict how patients would rate herbal remedies compared to conventional medicines. Disease states (hypertension, diabetes and asthma) did not influence the perception of efficacy. Further analysis showed that these demographic variables had no influence of patients' disclosure of herbal use to their physician.

**Table 5:** Comparative efficacy with herbal remedies with conventional medicine.

**Demographic factors**	**n**	**Rating of efficacy of Herbal Remedy Compared to Allopathic Medicines (%)**			
		Less Efficacious	Equally Efficacious	More Efficacious	Non Response
**Age Group**					
16 – 30	49	0 (0.0)	15 (30.6)	30 (61.2)	4 (8.2)
31 – 45	65	6 (9.2)	22 (33.8)	36 (55.4)	1 (1.5)
46 – 60	73	10 (13.7)	31 (42.5)	31 (42.5)	1 (1.4)
Over 60	76	10 (13.2)	29 (38.2)	34 (44.7)	3 (3.9)
Non Response	2				
		26 (9.8)	97 (36.6)	131 (49.4)	9 (3.4)
		NS			
**Gender**					
Male	71	9 (12.7)	22 (31.0)	40 (56.3)	0 (0.0)
Female	194	17 (8.8)	76 (39.2)	92 (47.4)	9 (4.6)
		26 (9.8)	98 (37.0)	132 (49.8)	9 (3.4)
		NS			
**Annual Income ($US)**					
< 3,999	132	14 (10.6)	42 (31.8)	70 (53.0)	6 (4.6)
4,000 – 9,999	91	10 (11.0)	34 (37.4)	45 (49.5)	2 (2.2)
10,000 – 14,999	13	0 (0.0)	7 (53.9)	5 (38.5)	1 (7.7)
> 15,000	9	1 (11.1)	3 (33.3)	5 (55.6)	0 (0.0)
Non Response	20				
		25 (9.4)	86 (32.5)	125 (47.2)	9 (3.4)
		NS			
**Years of formal education**					
None	19	2 (10.5)	8 (42.1)	9 (47.4)	0 (0.0)
≤ 7 years	121	16 (13.2)	45 (37.2)	56 (46.3)	4 (3.3)
> 7 years ≤ 12 years	94	5 (5.3)	33 (35.1)	53 (56.4)	3 (3.2)
> 12 years	25	3 (12.0)	10 (40.0)	10 (40.0)	2 (8.0)
Non Response	6				
		26 (9.8)	96 (36.2)	128 (48.3)	9 (3.4)
		NS			
**Ethnicity**					
African	69	5 (7.3)	23 (33.3)	38 (55.1)	3 (4.4)
Asian Indian	120	18 (15.0)	46 (38.3)	53 (44.2)	3 (2.5)
Mixed	74	3 (4.1)	27 (36.5)	41 (55.4)	3 (4.1)
Other	1	0 (0.0)	1 (100.0)	0 (0.0)	0 (0.0)
Non Response	1				
		26 (9.8)	97 (36.6)	132 (49.8)	9 (3.4)
		NS			

* NS- not a significant predictor variable in stepwise regression model

Sixteen out of 265 patients (6%) reported ever experiencing any herb-associated adverse effects. Three patients (3 of 16; 18.8%) reported gastrointestinal adverse effects with noni juice, two patients (12.5%) reported garlic-induced hypotension and another two patients (12.5%) reported diarrhoea after using unspecified herbs. Other cases include isolated incidents ranging from abdominal bloating and weakness caused by unspecified herbal 'colon cleansers' to ginger-induced hyperglycaemia. Garlic and ginger were also associated with adverse effects as they were among the most commonly used herbs in the sample.

Concomitant use of herbs and allopathic medicines was reported in eighty-one respondents (30.6%), with 57 of these (70.4%) reporting that they perceived that this combination "worked better". The most common herb-drug combinations were with flu medicines (10 of 81 patients; 12.4%) and garlic and anti-hypertensive drugs (10 of 81 patients; 12.4%). Nine patients (11.1%) also used lemongrass alongside paracetamol for fever. Only 3 patients (3.7%) using combined therapies reported ever experiencing adverse effects; and one patient continued even after the adverse effect presented because "it worked".

Respondents obtained herbs from multiple sources, however, the backyard was the most common (79.2%), 22 (7.5%) purchased from the market and only 2 patients (0.8%) from the 'bush doctor'.

## Discussion

Our findings highlighted the favourable perception of efficacy of herbal remedies held by users who accessed primary healthcare facilities throughout Trinidad. Most users of herbal remedies believed that this healthcare modality was an important and effective mode for health and wellness promotion and disease management, similar to a recent US study [11]. We also observed that most users believed that herbal remedies were either equally or more efficacious than conventional medicines, and in fact about half of the sample suggested that herbs were more efficacious than conventional medicines. Our findings, coupled with the high prevalence of herbal remedy use in Trinidad, demonstrated the overwhelming endorsement of this healthcare modality by patients accessing primary health care services on the island. Our results corroborated well with a recent survey conducted by Tindle and his colleagues [12] where a significant number of CAM users perceived that these therapies had greater efficacy than conventional allopathic medicines. In their study most respondents used conventional and CAM modalities concomitantly and rated the perceived efficacy of the combined use as greater than the individual modalities.

Our findings were also similar to other studies where patients' perception of the therapeutic efficacy of CAMs were assessed, in these studies more than half of CAM users perceived that the alternative healthcare modality was responsible for some noticeable improvement in physical or psychological well being [13-15]. Recently in Trinidad, 76.9% of physicians who reported the use of herbal remedies were satisfied with the outcome [7]. However, our present study results were markedly different than the mere 5.2% of urban Hispanics in the US who perceived that herbs were more efficacious than physician-prescribed allopathic medicines [16]. Our recent survey of asthmatics in Trinidad showed that although 30% of patients reported using herbal remedies to alleviate symptoms, none perceived that these remedies by themselves had greater efficacy than conventional medicines in relieving their symptoms [6]. However, 35% of herbal remedies users with moderate to severe asthma indicated that herbs when used concomitantly with conventional anti-asthmatic medication "worked better". It may be possible that that the rating of perceived efficacy of herbs is disease specific and correlated to concomitant use with allopathic medicines.

As elsewhere [17], most herbal users did so concomitantly with allopathic medicines, without the knowledge of their attending physician. They also indicated that concomitant herb-drug use was more beneficial than when either herb(s) or drug(s) were used alone. We propose that this practice could further consolidate the perception that this potentially dangerous practice is safe and encourage further 'uninformed' herb-drug concomitant use. Our reported herb-drug interaction rate of 3 out of 265 (1.1%) is relatively low, but this does not negate the grave importance of public awareness of the potential dangers of herb-drug combinations. Our study showed a relatively high incidence of herb-associated adverse effects in 16 out of 265 respondents (6%).

Previous studies have demonstrated that doctor-patient barriers to effective communication exist and these may contribute to nondisclosure. Although our analysis showed that none of the demographic variables influenced disclosure, other factors such as doctor-patient cultural differences, the GP's perception of patients of lower socioeconomic standing and the patients' perception of the GP's negative attitude towards herbal remedies may be affecting the extent of disclosure [18-20].

In our study, garlic was the most popular herb used and many clinically important garlic-drugs interactions have been identified [21]. Healthcare professionals are in a unique position to impart current evidence-based information to their patients on known deleterious herb-drug interactions [22-24]. In so doing informed decisions could be made in an open and non-judgmental environment regarding the use of herbs alongside allopathic medicines.

Although patients reported subjective improvements in physical and psychological well being this may in some cases be attributable to a placebo effect or the natural history of the disease, and may or may not translate into measurable clinical outcomes. Garlic was used by almost half of our sample and in a significant number for the therapeutic management of hypertension. A recent review highlights some of the cardiovascular benefits of garlic [25] and may support its use in our sample. Durak et al (2004) demonstrated that a standardized garlic extract increased antioxidant status, decreased plasma cholesterol levels, while decreasing blood pressure in hypertensive patients [26]. Garlic has also been shown to reduce oxidative stress [27,28], a presumed underlying cause of many diseases, and this would indirectly imply its usefulness as a preventative herbal medicine to promote health and wellness.

On the other hand, the perceived efficacy of a particular CAM may not translate into measurable clinical effects as was the case in a study involving chronic hepatitis C patients, where although most patients reported subjective improvements, none had normalized serum transamine activities after CAM use [9]. Many patients in our study reported the use of herbs for the common cold and cough, with perceived benefits; however, a recent systematic review for echinacea in common cold reported only marginal benefit [29]. These discrepancies underlie the imperative for well-designed randomised placebo-controlled trials to be conducted for the proper assessment of the clinical efficacy and safety of herbal medicines. These would determine the usefulness of these alternative modalities and the possible integration into mainstream conventional medical practice. These studies would inform physicians and patients alike on the measurable benefits and risks of specific herbal remedies.

An earlier report of patient characteristics at public health care facilities in Trinidad showed that almost half of these patients were unable to pay for conventional health care [30]. At the time of that study patients complained of poor pharmacy service and chronic unavailability of conventional medicines. Within the last few years the government of Trinidad and Tobago has introduced the chronic disease assistance program (CDAP) which has significantly improved the delivery of allopathic medicines to this disadvantaged sector of society. Nevertheless, this scenario of shortages of medicines over the years raises an interesting question as to how patients managed their health and disease in a compromised public health care system. Most patients in our sample sourced their herbs from their backyards and it may be possible that in actuality conventional medicines were added to the traditional practice of herbal use and not the other way around. Most patients reported using herbs on the advice of relatives and friends and this may point to some degree of traditional knowledge transfer.

Our survey was conducted at public primary health facilities throughout Trinidad and included only patients who confirmed their current or previous use of herbs; this selection bias may have introduced a number of limitations. The demographics of our sample demonstrate that patients accessing public primary healthcare were more likely to have low levels of formal education and income, high unemployment and more likely to be female. This correlates well with previous studies done on the island's public health care system [31,32]. Obviously, the sample demographics in our study do not reflect that of the wider Trinidadian society and our results cannot therefore be extrapolated to the general population. Since most patients in the sample did not purchase commercially prepared herbal supplements we were not able to capture the effect of corporate marketing influence in the purchase of commercially prepared herbal supplements in Trinidad.

Another possible limitation of the study could have been that some patients at the visited healthcare facilities may not have considered their customary use of "bush teas" as preventative and they may have been inadvertently excluded. Most patients attend primary healthcare facilities for either minor ailments or chronic disease management. This meant that the only patients with certain categories of diseases (such as hypertension, diabetes mellitus and asthma) or minor ailments were interviewed and that herbal remedies in the self-management of other more severe medical conditions such as HIV/AIDS and neoplasias would have been excluded. This, of course, was reflected in the responses obtained as most patients used herbs for maintenance of health and wellness and for minor ailments such as the common cold, cough and fever. Hypertension was the chronic disease most managed with herbal remedies in the sample.

Although we attempted to use quota sampling for the convenient and advantageous capturing of our sample, it may have been possible that interviewer bias may have been introduced by the non-random selection of patients, resulting in a sample that may not have been truly representative.

Notwithstanding these limitations our study results indicate that the high prevalence of herbal use in Trinidad may be attributable to the patients' underlying belief that herbs are efficacious and in some cases more efficacious than conventional medicines. This high prevalence of herbal use leaves us with little option but to accept that this modality would be around for some time and that important public health concerns must be urgently addressed. We therefore recommend that physicians at these public healthcare facilities become more knowledgeable about herbs so that they would be better able to communicate with their patients, especially with regard to their potential interactions with conventional medicines. We also support the conducting of well-designed randomised controlled clinical trials to establish the safety profile and efficacy of Caribbean medicinal herbs. These evidence-based studies would provide a platform for informed decisions by healthcare providers and more importantly the self-prescribing members of the public.

## Conclusion

In our study most herbal users perceived that herbs were efficacious, and in some instances, more efficacious than conventional medicines. Our findings support our hypothesis that this perception of efficacy is a major contributing factor sustaining the use of this healthcare modality. The growing body of evidence-based research in the form of randomized controlled clinical trials should direct the proper use of herbs and should continue to receive support to validate (or otherwise) efficacy and determine safety. Unfortunately, most indigenous Caribbean herbs are not well investigated and this points to the urgent need for biomedical investigations to assess safety profile and efficacy of popular medicinal herbs.

## Competing interests

The author(s) declare that they have no competing interests.

## Authors' contributions

YNC was the P.I. and was responsible for the study concept, development of methodology, coordinating of the research activities, analyzing the data, and writing the manuscript. LB, AB, M-JF, NG, and MJ were involved in methodological development, data collection and data input. AS was involved in methodological development and data collection. JM-G was involved in methodological development, data collection, data input and statistical analysis. All authors read and approved the final manuscript.

## Pre-publication history

The pre-publication history for this paper can be accessed here:


